# Comprehensive Analysis of *Berberis aristata* DC. Bark Extracts: In Vitro and In Silico Evaluation of Bioaccessibility and Safety

**DOI:** 10.3390/nu16172953

**Published:** 2024-09-02

**Authors:** Giovanna Rigillo, Giorgio Cappellucci, Giulia Baini, Federica Vaccaro, Elisabetta Miraldi, Luca Pani, Fabio Tascedda, Renato Bruni, Marco Biagi

**Affiliations:** 1 Department of Biomedical, Metabolic and Neural Sciences, University of Modena and Reggio Emilia, 41125 Modena, Italy; luca.pani@unimore.it; 2 Laboratory of Italian Society of Phytoterapy-SIFITLab, 53100 Siena, Italy; giorgi.cappellucci@unisi.it (G.C.); giulia.baini2@unisi.it (G.B.); fvaccaro@student.unisi.it (F.V.); elisabetta.miraldi@unisi.it (E.M.); marco.biagi@unipr.it (M.B.); 3 Department of Physical Sciences, Earth and Environment, University of Siena, 53100 Siena, Italy; 4 Department of Psychiatry and Behavioral Sciences, University of Miami, Miami, FL 33136, USA; 5 Department of Life Sciences, University of Modena and Reggio Emilia, 41125 Modena, Italy; fabio.tascedda@unimore.it; 6 Consorzio Interuniversitario Biotecnologie (CIB), 34148 Trieste, Italy; 7 Department of Food and Drug, University of Parma, 43124 Parma, Italy; renato.bruni@unipr.it

**Keywords:** berberine, safety, bioaccessibility, cell viability, gene expression

## Abstract

Berberine (BER) is an alkaloid found, together with other protoberberinoids (PROTBERs), in several species used in medicines and food supplements. While some herbal preparations containing BER and PROTBERs, such as *Berberis aristata* DC. bark extracts, have shown promising potential for human health, their safety has not been fully assessed. Recently, the EFSA issued a call for data to deepen the pharmacokinetic and pharmacodynamic understanding of products containing BER and PROTBERs and to comprehensively assess their safety, especially when used in food supplements. In this context, new data were collected in this work by assessing: (i) the phytochemical profile of 16 different commercial *B. aristata* dry extracts, which are among the most widely used preparations containing BER and PROTBERs in Europe; (ii) the In Vitro and In Silico investigation of the pharmacokinetic properties of BER and PROTBERs; (iii) the In Vitro cytotoxicity of selected extracts in different human cell lines, including tests on hepatic cells in the presence of CYP450 substrates; (iv) the effects of the extracts on cancer cell migration; and (v) the In Vitro molecular effects of extracts in non-cancer human cells. Results showed that commercial *B. aristata* extracts contain BER as the main constituent, with jatrorrhizine as main secondary PROTBER. BER and jatrorrhizine were found to have a good bioaccessibility rate, but they interact with P-gp. *B. aristata* extracts showed limited cytotoxicity and minimal interaction with CYP450 substrates. Furthermore, tested extracts demonstrated inhibition of cancer cell migration and were devoid of any pro-tumoral effects in normal cells. Overall, our work provides a valuable overview to better elucidate important concerns regarding botanicals containing BER and PROTBERs.

## 1. Introduction

**Figure 1:**
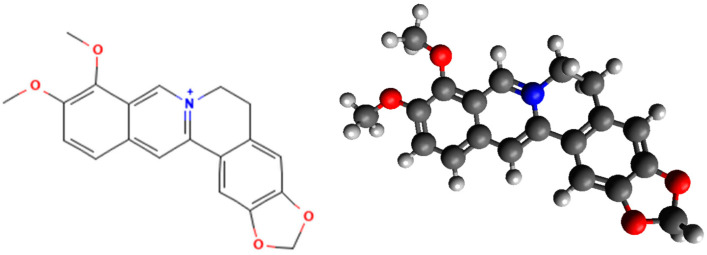
Berberine chemical structure in 2D and 3D (PubChem CID: 2353) (https://pubchem.ncbi.nlm.nih.gov/compound/Berberine, accessed on 22 August 2024).

Berberine (BER) is an isoquinoline alkaloid belonging to the category of protoberberines, including berberrubine, thalifendine, demethyleneberberine, coptisine, jatrorrhizine, columbamine, palmatine, and epiberberine. These compounds share a common molecular moiety, characterized by an aromatic quaternary ammonium nitrogen, a bright yellow color, and water solubility [1] (Figure 1). 

BER is a characteristic compound with biological activity found in several plant species with a well-established use in traditional Asian medicine, such as those in *Berberis*, *Coptis*, *Phellodendron*, *Corydalis*, and *Xanthorhiza* genera or in *Tinospora sinensis* (Lour.) Merr. and *Coscininium fenestratum* (Goetgh.) Colebr., but it also occurs in medicinal species known in American and European Western medicine, such as *Hydrastis canadensis* L., *Chelidonium majus* L., or *Papaver* spp., as well as in Siberia (*Thalictrum flavum* L.) and in Africa (*Jateorhiza palmata* (Lam.) Miers and *Annickia chlorantha* (Oliv.) Setten & Maas) [2]. In herbal preparations obtained from these species, other compounds may contribute to the biological activity of BER, in particular the alkaloids chemically related to BER, such as protoberberinoid derivatives (PROTBERs) [3].

To date, in the European Union (EU), an official monograph on the medicinal use of these species has not been produced by the European Medicines Agency (EMA), while a *Berberis aristata* DC. stem monograph was added to the European Pharmacopoeia in 2022, being the most commonly used BER-containing species used in food supplements [4,5].

BER and BER-containing herbal preparations from *B. aristata* have displayed a plethora of biological effects, as witnessed by numerous clinical trials. A high-quality meta-analysis [3] reports that most clinical trials concern cardiovascular system disorders (atherosclerosis and lipid profile) [5,6,7,8], inflammation [9,10], glycemia [11,12,13,14], gastrointestinal health [15,16], and cancer [17,18,19]. Evidence indicates that BER shows beneficial properties in conditions of dysregulation or alteration of physiological processes, displaying various activities such as lipid and glycemic regulation, anti-inflammatory, antioxidant and antiproliferative activities, and gut microbiota modulation [20]. The biological activity of BER has been associated with multiple molecular mechanisms involving different cell signaling pathways; PKA, p38 MAPK, Wnt/β-catenin, AMPK, RANK/RANKL/OPG, PI3K/Akt, NFAT, NF-κB, Hedgehog, low-density lipoprotein (LDL) receptor expression, reactive oxygen species (ROS), and nitric oxide (NO) production have been described as molecular targets of BER in different conditions regarding sugars and lipids metabolism, cardiovascular, gastrointestinal, musculoskeletal, and the central nervous systems, as well as cancer and inflammation [21,22,23,24,25,26].

Potential adverse events could be linked to the biological activity of BER and PROTBERs; therefore, the safety of BER, PROTBERs, and BER-containing herbal preparations has been carefully considered. 

In terms of pharmacokinetics, BER and PROTBERs show different bioavailability: BER is a substrate for P-glycoprotein (P-gp) and less than 1% of an oral dose was found to be bioavailable [27]; also, jatrorrhizine is a P-gp substrate and it showed a poor bioavailability [28,29]. Other PROTBERs have a higher bioavailability, as demonstrated by in vivo studies for epiberberine [30], palmatine [31], berberubbine [32], coptisine, and columbamine [33]. Despite the lack of proper knowledge on cellular bioavailability, it is well known that after intestinal absorption, BER and PROTBERs are rapidly metabolized by hepatic CYP450 enzymes. Many studies have primarily described BER metabolism, reporting that the CYP450 1A2, 3A4, 2D6, and 2C9 isoforms are mainly involved [34], while few have studied other PROTBERs and their interaction with different CYP450 isoforms, with the exception of jatrorrhizine, metabolized by CYP1A2 [35], and palmatine by CYP2D6 [36]. It is plausible that CYP1A2, CYP3A4, and CYP2D6 have a key role in the metabolism of many PROTBERs; thus, drug–herbal product interactions should be considered when BER, PROTBERs, and BER-containing herbal preparations are used.

Given the complexity of the topic and the well-established and growing consumption of BER-containing herbal preparations, especially in food supplements, in 2023 the European Food Safety Authority (EFSA) opened a “call for data” to obtain relevant updates on the safety assessment of BER-containing herbal preparations used in food supplements [37].

Within this context, this study aims to provide new experimental-based evidence to fill some pivotal gaps concerning the chemical and pharmacokinetic aspects, and biological safety of berberine. To accomplish our aim, this research was structured into seven steps:

(1) the phytochemical characterization of sixteen different commercial raw BER-containing ingredients, used in food supplement formulation, through the development of an efficient HPLC-DAD method to quantify BER and PROTBERs;
(2) In Vitro evaluation of digestive stability and bioaccessibility, and In Silico investigation of pharmacokinetic properties of BER and PROTBERs;
(3) prediction of potential BER-related targets by bioinformatic analysis;
(4) assessment of cytotoxicity in In Vitro human intestinal (Caco-2), hepatic (HepG2), gastric (AGS), and kidney (HEK293) cell lines, and of BER’s impact on hepatic cell viability in the presence of CYP450 substrates;
(5) evaluation of BER’s effects on cell migration ability of colorectal carcinoma cells (Caco-2);
(6) investigation of potential oxidative stress of BER in non-tumoral kidney cells (HEK293) by the dosage of ROS;
(7) evaluation of the transcriptional effects of BER on the main target genes involved in the regulation of cell cycle, cell growth, and neoplastic transformation, as well as on oncogenes.

## 2. Materials and Methods

### 2.1. Plant Material

Sixteen different extracts used as raw ingredients in Italy for food supplement preparations were selected from the international business-to-business market. According to their declared content, they were divided into two groups: eight *B. aristata* bark extracts with a declared content of 97% BER *w*/*w* dry basis (db) as BER hydrochloride, and the remnant with a declared content of 85% BER *w*/*w* db. Samples were kindly supplied by EHPM (Brussels, Belgium), Viatris (Monza-Brianza, Italy), Giellepi (Seregno, Monza-Brianza, Italy), and Vivatis (Gallarate, Varese, Italy).

### 2.2. Phytochemical Analysis

The quantification of BER and PROTBERs in the examined extracts was determined by analytical techniques and measured by calculating values as BER hydrochloride on the dry basis (db) of the extract (% *w*/*w* db). All samples were dissolved in ultra-pure water (1 mg/mL) and analyzed by using an HPLC-DAD Shimadzu Prominence LC 2030 3D instrument. A Bondpak^®^ C18 column, 10 µm, 125 Å, 3.9 mm × 300 mm (Waters Corporation, Milford, MA, USA), was used as the stationary phase. The mobile phase was composed of water with 0.1% *v*/*v* formic acid (A) and acetonitrile with 0.1% *v*/*v* formic acid (B). The following method was used: B from 45% at 0 min and a linear increase to 55% at 9 min, then 45% at 10 min, holding the same percentage until the end, 11 min. Flow rate was set at 0.900 mL/min; column temperature was 30 °C. The chromatogram was recorded at 346 nm. BER hydrochloride (Merck KGaA, Darmstadt, Germany) (10–0.01 μg in column) was used to build the calibration curve. In herbal preparations, BER was identified and quantified by using the specific external standard, whereas other PROTBERs were identified according to the literature and their UV–vis spectra [31]; PROTBERs were quantified and expressed as BER hydrochloride.

Given the homogeneous phytochemical profile of the extracts, subsequent bioactivities were determined using two different *B. aristata* bark extracts with a declared content of 97% BER *w*/*w*, and two *B. aristata* bark extracts with a BER content of 85% *w*/*w*, namely, A85, D85, A97, and G97 (Table 3 in Section 3.1).

### 2.3. In Vitro Bioaccessibility Assessment 

The test was conducted according to Governa et al., 2022 [38] and the validated INFOGEST protocol [39], with some modifications. In detail, 20 mg of each extract were added to 20 mL of simulated gastric juice, containing pepsin from porcine gastric mucosa (300 UI/mL, Merck) and NaCl (10 mg/mL); the pH of the solution was adjusted to 1.7 using HCl. Samples were incubated for 2 h at 37 °C with shaking. Then, pancreatin from porcine pancreas (activity equivalent to 4× U.S.P., 10 mg/mL, Merck) and a bile salt mixture (20 mg/mL, Merck) were added, and the pH was increased to 7.0 by adding NaHCO_3_ (15 mg/mL, Merck) to simulate the intestinal environment. Intestinal digestion was carried out for 2 h at 37 °C with shaking. Samples were then filtered and immediately used for further analysis, performed according to the HPLC-DAD method described above. The bioaccessibility rate of each compound was calculated as the % of its recovery after digestion, compared to the initial amount. Two independent experiments were performed.

### 2.4. In Silico Pharmacokinetic Analysis and Target Prediction 

Computational analysis of pharmacokinetic characteristics of BER and PROTBERs present in the most common food supplements marketed in the EU was performed using the SwissADME© web tool, a free platform created and developed by the Molecular Modeling Group of the SIB, Swiss Institute of Bioinformatics. According to their chemical–physical characteristics, compound similarities, and provided data, the trained algorithm estimates compounds for ADME (absorption, distribution, metabolism, and excretion) properties, physical chemistry, drug likeness, pharmacokinetics, and medicinal chemistry properties [40]. Target prediction was performed by using GeneCards^®^ suite, a web tool developed to obtain results with functionality and relevance scoring, allowing for the combination of query terms and providing relevant literature, which the match is based on [41]. In this work, SwissTargetPrediction, another web tool of the SIB, was used, as well as SEA (Similarity Ensemble Approach). SEA predicts the biological targets of a compound based on its resemblance to ligands annotated in reference databases and relates proteins by their pharmacology by aggregating chemical similarity among entire sets of ligands; SEA also scores Tanimoto similarity calculations based on compound annotations derived from ChEMBL [42]. 

### 2.5. In Vitro Cell Culture and Treatment 

Human colorectal (Caco-2), hepatic (HepG2), gastric (AGS), and kidney (HEK293) cell lines were cultured in DMEM supplemented with 10% fetal bovine serum (FBS), 1% glutamine, and 1% penicillin/streptomycin antibiotics. Media and material for cell cultures was supplied by Merck. Cells were maintained under a humidified atmosphere of 5% CO_2_ at 37 °C. Cells were treated with two representative samples based on the results obtained from chemical analysis: one *B. aristata* bark extract with 97% BER (as berberine hydrochloride) *w*/*w*, composed of two pooled extracts containing BER 97% (B97%) and one *B. aristata* bark extracts with 85% BER content *w*/*w*, composed of two pooled extracts containing BER 85% (B85%). The control group received phosphate-buffered saline (PBS). The treatments were carried out at various time points, as indicated in each section, for further analysis.

### 2.6. Cytotoxicity Assay

Caco-2, HepG2, HEK293, and AGS cells were seeded in 96-well plates and cultured for 24 h. Cells were treated with B97% and B85% extracts at 10, 20, 50, 100, and 200 μg/mL for 4 and 24 h. After the treatment, the medium was removed, and cells were incubated with fresh medium in the presence of 10% *v*/*v* CCK-8 (Cell Counting Kit, Merck) [43,44]. The assay is based on the reduction of a water-soluble tetrazolium salt WST-8 operated by cell dehydrogenases that lead to formazan production, soluble and orange-colored; the absorbance of the formazan dye is measured at 450 nm with a microplate reader VICTOR^®^ Nivo™3s (Perkin-Elmer, Waltham, MA, USA). Cell viability in treated groups was compared to untreated cells (control). Two independent tests were performed (*n* = 8).

### 2.7. Cytotoxicity Assay in Presence of CYP450 Substrates

Hepatic HepG2 cells were seeded in 96-well plates and cultured for 24 h. Firstly, cell viability was tested for the CYP450 substrates, specifically phenacetin (CYP1A2), dextromethorphan (CYP2D6), and triazolam (CYP3A4) at the concentrations of 0.1, 1, 10, and 50 μg/mL for 24 h (all drugs were purchased from Merck). After evaluating non-cytotoxic concentrations, HepG2 cells were seeded in 96-well plates and cultured, then treated with B97% and B85% at 50 or 100 μg/mL in the presence of phenacetin, dextromethorphan, or triazolam at 20 μg/mL for 24 h. Cell viability was assessed by means of the CCK-8 kit as described above.

### 2.8. Dosage of Intracellular Reactive Oxygen Species (ROS) Level

ROS production was quantified using 2′,7′-dichlorodihydrofluorescein diacetate (H2-DCF-DA, Thermo Fischer Scientific, Waltham, MA, USA) [45]. HEK293 cells were seeded in 96-well plates and cultured for 24 h. Cells were treated with B97% and B85% extracts at 10, 20, and 50 μg/mL for 24 h. H_2_O_2_, 0.5 mM, was used as a positive control of ROS production. After the treatment, the medium was removed, and cells were washed twice with PBS, then incubated with a 50 μM H2DCF-DA solution for 45 min at 37 °C. In the presence of ROS, the reagent H2DCF-DA was converted in a fluorescent adduct, dichlorofluorescein (DCF). DCF fluorescence intensity was measured at an excitation of 485 nm and emission of 535 nm, using a Multilabel Plate Reader VICTOR^®^ Nivo™3s (Perkin-Elmer). Two independent experiments with four replicates (*n* = 8) were performed.

### 2.9. Migration Assay 

Caco-2 cells (5 × 10^5^) were seeded into 6-well cell culture plates and allowed to grow to 70–80% confluence as a monolayer [43,46,47]. The monolayer was gently scratched across the center of the well with a sterile 1 mL pipette tip. A second scratch was performed perpendicular to the first, creating a cross in each well. After scratching, the medium was removed, and the wells were washed twice in PBS solution. Fresh medium containing 5% *v*/*v* of heat-inactivated FBS and B97% or B85% at 10 or 100 μg/mL, respectively, were added to each well. Images were obtained from the same fields immediately after scratching (t_0_) and after 6, 24, 30, and 48 h using a Leica DMIL microscope, and analyzed using ImageJ software version v1.54j by manually selecting the wound region and recording the total area. The experiments were conducted in triplicate, and two fields were analyzed for each replicate (*n* = 4). Untreated scratched cells represented the control. The percentage of scratching gap was calculated using the following formula: [Wound area t)/Wound area t_0_] × 100.

### 2.10. Total RNA Extraction, Reverse Transcription, and Real-Time PCR

HEK293 cells were seeded in 6-well plates at the density of 2 × 10^6^ cells/well and cultured for 24 h. Cells were stimulated with B97% and B85% at 10 and 20 µg/mL for 24 h. RNA extraction and DNAse treatment were performed as previously described [31,48] by using Ripospin II mini-Kit (#314–150, GeneAll, Seoul, Republic of Korea), according to the manufacturer’s protocol. For cDNA synthesis, one microgram of RNA was retrotranscribed with PrimeScript RT Reagent Kit (#RR037A, Takara Bio, Shiga, Japan) and RT-qPCR was performed by CFX Connect Real-Time PCR machine (Bio-Rad Laboratories, Hercules, CA, USA), using SsoAdvanced Universal SYBR Green Supermix (Bio-Rad Laboratories) and specific forward and reverse primers at a final concentration of 300 nM (Table 1). Cycle threshold (Cq) value was determined by the CFX maestro software version 2.3 (Bio-Rad Laboratories), and mRNA expression was calculated by the ΔΔCt method and normalized to the mean of rps18-rpl13a genes as an endogenous control. For gene expression analysis, endogenous control mRNA levels were not affected among treatments (*p* > 0.05, one-way analysis of variance: ANOVA).

### 2.11. Statistical Analysis

Data were presented as mean ± standard deviation (SD). Statistical analyses were performed using the unpaired Student *t*-test or one-way analysis of variance (ANOVA) (with *p* < 0.05 significance level) as appropriate, followed by Dunnet post-hoc tests for multiple comparison. Analyses and graphs were conducted and composed by using GraphPad Prism 10.1 (San Diego, CA, USA).

## 3. Results

### 3.1. Chemical Analyses of Extracts Containing Berberine and Protoberberinoids

Chemical analysis of sixteen different marketed herbal preparations containing BER and PROTBERs were performed, specifically extracts from *B. aristata*, namely *B. aristata* bark extracts with a declared content of BER of 97% *w*/*w* db, and *B. aristata* bark extracts with a declared content of BER of 85% *w*/*w* db. A new method developed on purpose was used, providing a robust and reliable performance for the extracts under investigation containing BER and PROTBERs (Table 2). 

The method allowed us to perfectly identify and quantify BER (as BER hydrochloride) in all samples analyzed at the retention time (RT) of 5.7 min (Table 3).

**Figure 2:**
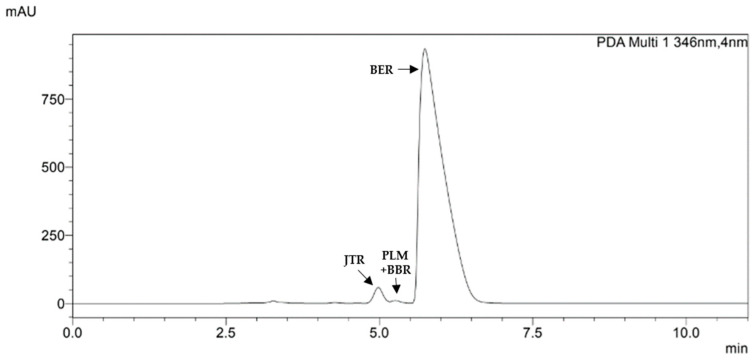
HPLC chromatogram of a representative *Berberis aristata* DC. bark extract recorded at 346 nm. The main peak (retention time 5.7 min) is related to berberine (BER); at 5.0 min, there is jatrorrhizine (JTR). Among these two molecules, at 5.3 min, two partially overlapped peaks are present, attributed to berberrubine (BBR) and palmatine (PLM).

We also identified jatrorrhizine in all the samples (RT = 5.0 min), as well as palmatine (RT = 5.3 min), in agreement with previously published works [49] and with the characteristic UV–vis spectra. A partial overlapping of palmatine with another minor PROTBERs was noted, and thanks to the collaboration of external labs and by using a mass spectrometer, we identified this PROTBER as berberrubine. In many samples, we found other secondary PROTBERs through their maximum absorbance at 344–347 nm, but the sum of these compounds expressed as BER hydrochloride was confirmed to be under the limit of quantification (LOQ) (Figure 2).

Summarizing, in all samples, we found BER as a major compound, followed by jatrorrhizine, always present, and palmatine at low concentrations, often accompanied by berberrubine (Table 4). The overall qualitative and quantitative data proved the extracts were true to the label for both the 97% and 85% groups, with a maximum discordance of +8.2% for C85 and −5.8% for G85. The higher variability in BER content in samples labeled with BER 85% compared to those labeled with BER 97% was noted; the reason for this are not known to date, but we could generally attribute it to differences in the method of extract preparation.

Contrarily to what emerged for other botanicals, the quality of *B. aristata* extracts available in the raw ingredients marketplace is reliable [50]. This evidence, however, is seemingly in contrast with recent investigations in which only 56% of 18 *Berberis*-derived food supplements were true-to-the-label [27]. It must be specified in this regard that our samples were crude extracts available in the business-to-business ingredients market rather than retail, formulated food supplements.

Given the homogeneous phytochemical profile, for subsequent tests, two *B. aristata* extracts with a declared content of 97% of berberine hydrochloride db and two extracts of *B. aristata* with a declared content of 85% of berberine hydrochloride were chosen randomly (A85, D85, A97, G97) and pooled to obtain the samples B85 and B97, whose chemical composition is reported in Table 5.

### 3.2. In Vitro Bioaccessibility Assessment of Berberine and Protoberberinoids 

**Figure 3:**
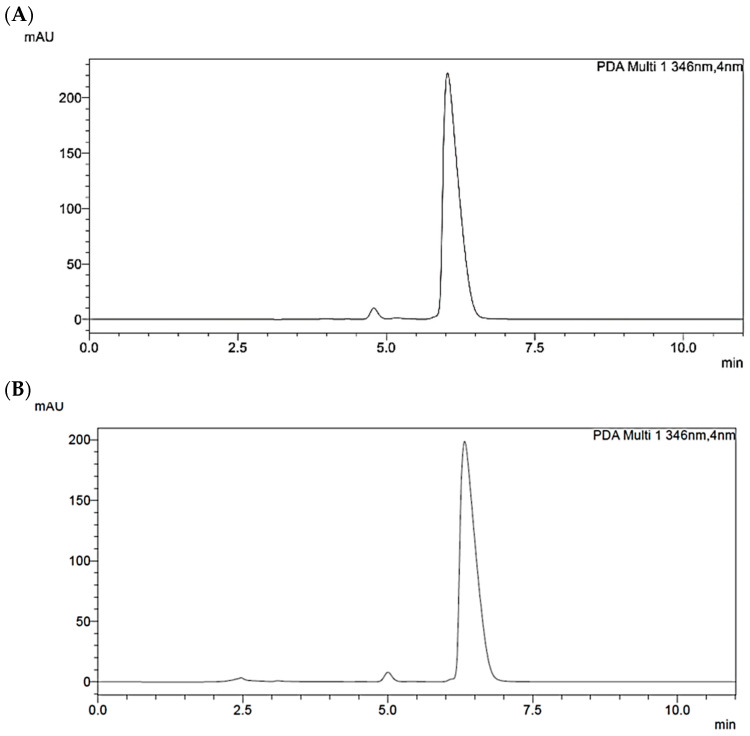
Representative HPLC chromatogram of *Berberis aristata* DC. bark extract containing 85% of berberine hydrochloride dry basis before (**A**) and after (**B**) simulated digestion, recorded at 346 nm.

The assessment of simulated digestion on the different *B. aristata* extracts examined allowed us to recover BER and jatrorrhizine in all samples (Table 6). The post-digestive presence of bile salts and enzymes partly hid the minority part of berberrubine and palmatine in some replicates; thus, we decided not to report these PROTBERs in the assessment of bioaccessibility. We could verify that isoquinolinic alkaloids underwent minimal degradation; indeed, the post-digestive bioaccessibility rate was >95% for BER and 84–88% for jatrorrhizine, with low differences in different samples (Table 6). BER was adequately soluble in hydrophilic gastrointestinal digestive fluids, and it was not affected by the activity of digestive enzymes. Figure 3A,B shows the stability obtained from the analysis of a representative extract before and after simulated digestion. 

### 3.3. In Silico Pharmacokinetic Analysis and Target Prediction of Berberine and Protoberberine Derivatives

After testing the bioaccessibility of BER and PROTBERs, we intended to study other pharmacokinetic features of BER, jatrorrhizine, berberrubine, and palmatine that are stable and present at detectable levels in the most used herbal preparations containing BER and PROTBERs.

**Figure 4:**
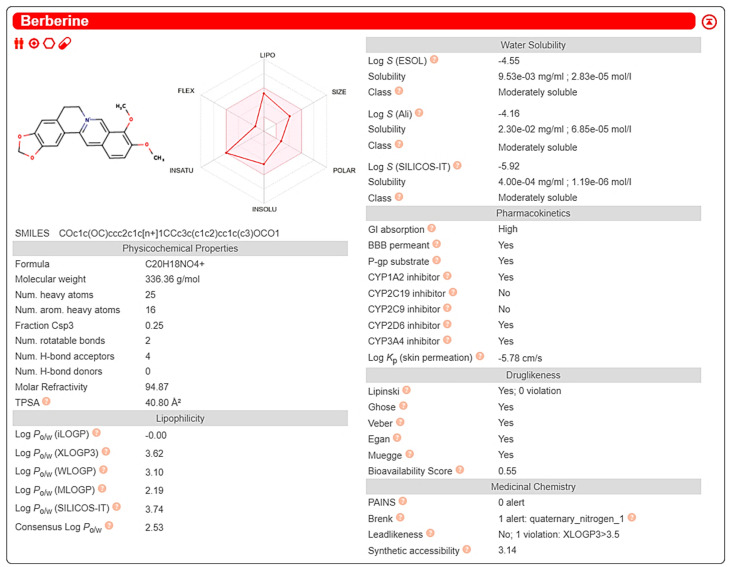
Computational analysis of berberine pharmacokinetic properties by means of SwissADME tool (http://www.swissadme.ch/, accessed on 20 January 2024).

By means of SwissADME^®^ tools, we verified that BER (both in neutral and in hydrochloride forms) has good potential to be absorbed by intestinal epithelium, but being a P-gp substrate explains data referring to its poor bioavailability [30,51]. Moreover, the computational prediction confirmed that BER is a substrate for the following CYP450 isoforms: 1A2, 3A4, and 2D6 (Figure 4). 

Jatrorrhizine and berberrubine were shown to share almost all pharmacokinetic characteristics with BER (Supplementary Figure S1A,B). As observed for BER, jatrorrhizine, berberrubine, and palmatine resulted in a P-gp, CYP2D6, and CYP3A4 substrate, but not CYP1A2; moreover, palmatine was the least soluble PROTBER among those examined (Supplementary Figure S1C).

**Figure 5:**
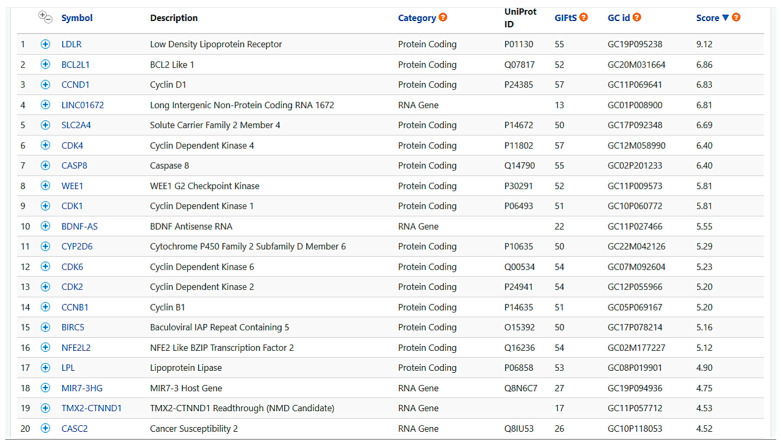
The best score for analysis of target prediction for berberine, as predicted by GeneCards tool (https://www.genecards.org/, accessed on 23 January 2024). Predicted targets are very heterogeneous but mainly refer to the most studied biological activities of berberine.

We used the GeneCards tool to collect known targets for BER and the other PROTBERs investigated here, and we found a total of 371 targets for BER, 6 for jatrorrhizine, 11 for berberrubine, and 16 for palmatine. Interestingly, the overlapping of targets found for the 4 compounds produced 373 results, thereby demonstrating that almost all targets known for BER are shared with other major PROTBERs of *Berberis* spp. (Figure 5).

We observed that the main targets identified by GeneCards were related to low-density lipoprotein receptor (LDLR) and antiproliferative activity, for which BER activity is known [52,53,54,55]. Other targets were related to CYP450 interaction (CYP2D6) and anti-neuroinflammatory activity (BDNF, NFE2), as already reported in pre-clinical studies [56] (Figure 5).

The interaction between BER and jatrorrhizine mainly underlined the activity towards neuroprotective-related targets (BDNF and acetylcholinesterase, ACHE) and on cell cycle regulatory factors (Supplementary Figure S2A).

Differently, the analysis of the interaction between BER and berberrubine highlighted the anti-inflammatory activity of berberrubine [57] (Supplementary Figure S2B).

As regards the combination BER with palmatine, we found that for some newly emerged targets, such as those related to cell cycle regulation and antiproliferative activity, corroborative data emerged from an In Vitro study on cancer cells [58,59,60,61]; moreover, BDNF and cholinesterase (BCHE) emerged as neuro-targets, and some important antioxidant targets such as catalase (CAT) and superoxide dismutase (SOD) were shared by BER and palmatine, as well as the xenobiotic toxicity modulator aryl-hydrocarbon receptor (ARH) [62]. CYP1A(1-2) resulted to be modulated by BER and palmatine (Supplementary Figure S2C).

Swiss and SEA target prediction, set as free search, without organ or signaling restrictions, confirmed that BER has a strong probability to interact with CYP2D6, as already described, but also with CYP1B1 and CYP1A2 (predicted by STP and SEA, respectively) (Supplementary Figure S3A). Both prediction tools also indicated acetylcholineesterase and cholinesterases (ACHE, BCHE) as targets, and the interaction with Ras-related C3 botulinum toxin substrate 1 (RAC1), a member of Rho GTPase (Supplementary Figure S3A). These predictions, only in part already known, may explain why BER is currently considered in the field of neurodegenerative disorders [63], vascular system [64], and metabolism regulation [24].

Regarding jatrorrhizine, the tools predicted affinity with medium or low scores, but ACHE was the most plausible target for this PROTBER. STP and SEA shared the prediction of RAC1, involved in metabolism, and cell division control protein 42 homolog (CDC42), a regulator of the cell cycle (Supplementary Figure S3B).

Prediction scores for berberrubine were the worst, being weak only for humans; STP predicted with a medium or low score some targets already considered for BER, such as RAC1 and CDC42 (Supplementary Figure S3C).

As observed for BER, palmatine interacted with ACHE, as the data experimentally confirmed [65]. Other targets such as 5-HTRB2, BCHE, ADRA2, CHRM1, SIGMAR1, and CYP2D6 were predicted with a medium score by SwissTarget, and RAC1 by SEA with a low score (Supplementary Figure S3D). 

### 3.4. In Vitro Cytotoxicity Evaluation of Berberis aristata Bark Extracts 

To assess the biological safety of BER and PROTBER alkaloids, we investigated the biological impact of both extracts (B97% and B85%) examined so far. Specifically, we evaluated the possible cytotoxic effects of BER- and PROTBERs-containing preparations by performing a cell viability assay on an In Vitro model of different human cell lines: intestinal (Caco-2), hepatic (HepG2), gastric (AGS), and renal (HEK293). Cells were treated with B97% and B85% at different concentrations (10, 20, 50, 100, and 200 µg/mL) at two time points, a short- (4 h) and a long-term (24 h), to simulate the exposure time of different organism systems (stomach, intestine, liver, and kidney) and cell types to BER and PROTBERs. The results obtained by the cell viability assay allowed us to determine the IC_50_ (half-maximal inhibitory concentration) value for each cell line at both time points of treatment. After 4 h of treatment, in all cell lines, the IC_50_ values were over 100 µg/mL; specifically, for the hepatic (HepG2) and gastric (AGS) cells, the IC_50_ values exceeded 200 µg/mL (Table 7). Similarly, after 24 h of treatment, the IC_50_ values were still higher than 100 µg/mL despite the increasing time of treatment. Of note, differences in the extracts tested were very low, demonstrating a similar cell impact of the different *B. aristata* extracts studied here. Differences in cell viability were observed in the various cell line models, but they could be considered non-significant given the high IC_50_ value obtained (Table 7). These data suggested a negligible cytotoxic impact of BER-containing herbal preparations, providing important outcomes on the safety of the use of BER and PROTBERs contained in the most common marketed food supplements.

### 3.5. In Vitro Cytotoxicity Evaluation of Berberis aristata Bark Extracts in Presence of CYP450 Substrates

Considering the hepatic metabolism of BER and PROTBERs mediated by CYP450, our investigation included the possible toxicological interactions with other drugs known to be substrates of CYP450 isoforms.

To address this aspect, we performed a cell viability assay on HepG2 cells treated with both B97% and B85% extracts at the highest concentrations of 50 and 100 µg/mL in the presence of three main CYP450 substrates, like phenacetin (P), dextromethorphan (D), and triazolam (T). 

**Figure 6:**
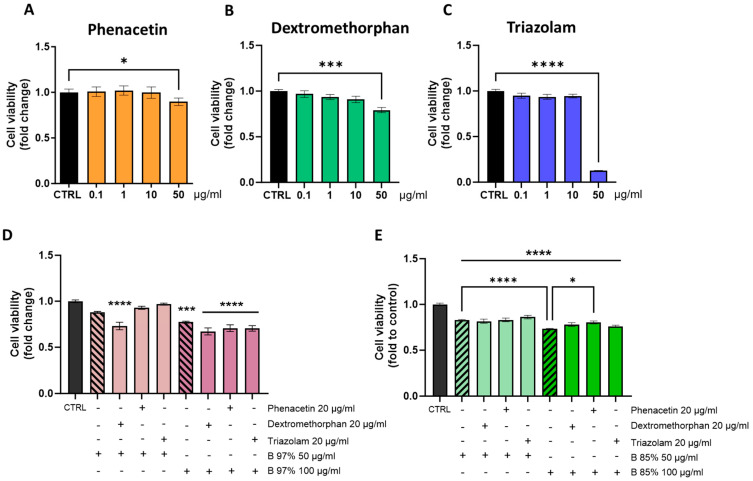
Cell viability assay on HepG2 cells after 24 h of treatment with different concentrations of (**A**) phenacetin, (**B**) dextromethorphan, and (**C**) triazolam. Then, HepG2 cells were co-treated for 24 h with phenacetin, dextromethorphan, or triazolam at 20 µg/mL and B97% at 50 and 100 µg/mL (**D**) or B85% at 50 µg/mL (light colour) and 100 µg/mL (dark colour)(**E**), respectively. The column with the striped pattern represents the treatment with only the extract. Each column represents mean ± SEM. Data were analyzed by one-way analysis of variance followed by Dunnet post-hoc: *** *p* < 0.001 and **** *p* < 0.0001 vs. CTRL; **** *p* < 0.0001 vs. B85% 50 µg/mL; * *p*< 0.05 vs. B85% 100 µg/mL; *n* = 4.

The selection of concentrations derived from the cytotoxicity tests described in 3.4 were intended to create a stress condition that could better assess the possible effects of interaction with other drugs at the cytotoxic level. Firstly, to identify the non-toxic concentration of P, D, and T to use in the co-treatment, we tested HepG2 cell viability treated with different concentrations of P, D, and T (0.1, 1, 10, and 50 µg/mL) for 24 h. Statistical analysis revealed that all three drugs showed no impact on cell viability up to 10 µg/mL compared to the control group (Figure 6A–C). At the dose of 50 µg/mL, P, D, and T differently affected the viability of hepatic cells: P and D reduced cell viability by about 10% and 20%, respectively (one-way ANOVA: P = *p* < 0.05; D = *p* < 0.001 vs. CTRL), while T showed the most significant impact by reducing cell viability of about 90% with respect to controls (*p* < 0.0001 vs. CTRL) (Figure 6C). Based on these data, we carried out following analysis by treating hepatic cells with 20 µg/mL of P, D or T in association with B97% or B85% at 50 or 100 µg/mL, respectively. The results showed a non-significant effect of B97% at 50 µg/mL on HepG2 cell viability; the association with P and T at 20 µg/mL did not cause changes in cell viability when compared to the control group, while the effect of BER was slightly worsened by the presence of D, resulting in a reduction in cell viability of up to 30% (one way ANOVA: *p* < 0.0001 vs. CTRL) (Figure 6D). The treatment with B97% at 100 µg/mL significantly affected cell viability compared to controls (*p* < 0.001 vs. CTRL), but it was not significantly altered by the co-presence of the three drugs (Figure 6D). Regarding B85%, the exposure of HepG2 cells to the sample alone slightly reduced the cell viability (−17% compared to control) that did not undergo alterations in the presence of P, D, or T when compared to the control cells (Figure 6E). The 24 h treatment of hepatic cells with B85% at 100 µg/mL significantly reduced the cell viability by about 30% with respect to the controls (*p* < 0.0001 vs. CTRL). Also, in this case, the association with all drugs did not change the effect of BER (Figure 6E); indeed, the co-presence of P seemed to improve the impact of B85% on cell viability (*p* < 0.05 vs. B85% at 100 µg/mL).

These results showed that, in association with P, D, or T, the low cytotoxicity of the most common BER-containing extracts remained unchanged. Overall, these findings provided preliminary data on toxicological aspects related to the interaction of B97% and B85% extracts with drugs known to be CYP450 substrates. 

### 3.6. Effect of Berberis aristata Bark Extracts on Cell Migration

In addition to assessing the effects on cell viability, the investigation of the safety of herbal preparations containing BER and PROTBERs proceeded by analyzing cell migratory movement by means of the wound-healing assay. This is a commonly used method to determine the effects of compounds on changes in 2D cell migration properties, relating in particular to cancer cells.

**Figure 7:**
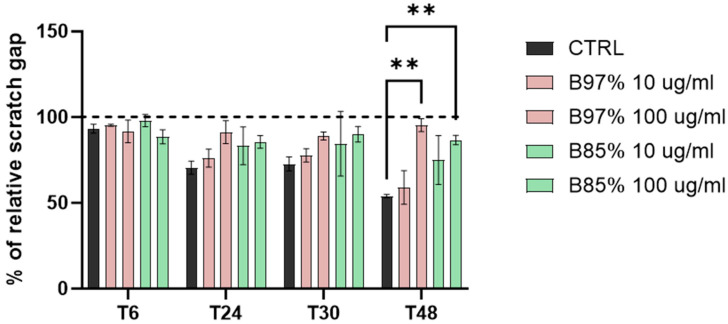
Wound-healing assay on Caco-2 cells treated with B97% and B85% (10 and 100 µg/mL) for 48 h or untreated (CTRL). Dashed line represent the 100% of wound area. Each column represents mean ± SEM. Data were analyzed by one-way analysis of variance: ** *p* < 0.01 vs. CTRL; *n* = 4.

With this purpose, we explored the migratory activity of human colorectal cancer cells (Caco-2) following treatment with B97% and B85% at both low concentration (10 µg/mL) and high concentration (100 µg/mL) for 48 h. The relative scratch gap was monitored over time and measured. Data showed a statistically significant effect of both B97% and B85% at the dose of 100 µg/mL in decreasing the migration ability of Caco-2 cells (about 40% B97% and 33% B85%) at the end of the treatment compared to control cells (one-way ANOVA: ** *p* < 0.01 vs. CTRL) (Figure 7). These results suggested a functional activity of tested extracts at 100 µg/mL in decreasing the cell migration of intestinal tumor cells. 

### 3.7. In Vitro Evaluation of ROS Production in Normal Kidney Cells Treated with Berberis aristata Bark Extracts 

The assessment of the safety of herbal preparations containing BER and PROTBERs also took into consideration the possible effects of oxidative stress of both B97% and B85% extracts in non-tumoral human cells. For this reason, ROS production was evaluated in normal renal cells (HEK293) treated for 24 h with B97% and B85% extracts at different concentrations (10, 20, and 50 µg/mL); H_2_O_2_ was used as a positive control.

**Figure 8:**
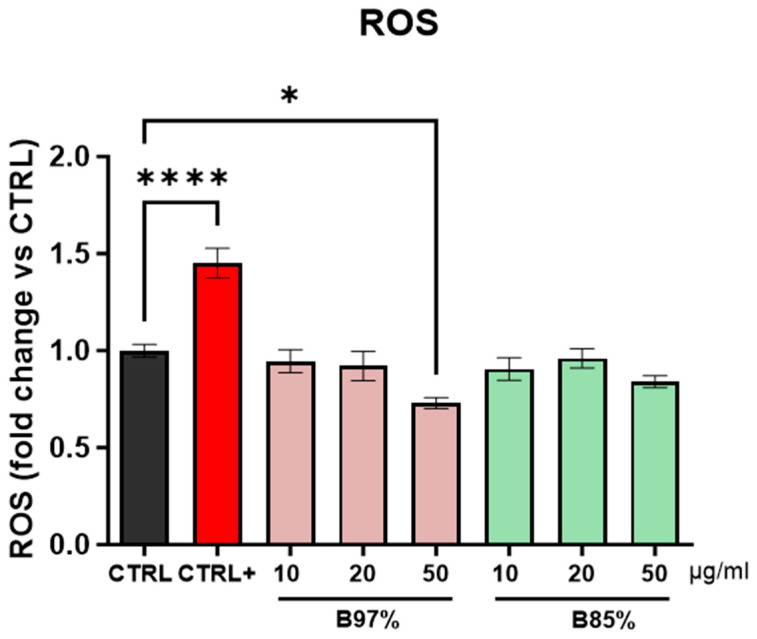
Dosage of intracellular ROS in HEK293 cells treated with B97% and B85% at the concentrations of 10, 20, and 50 µg/mL for 24 h. H_2_O_2_ (500 μM) was used as positive control (CTRL+). Each column represents mean ± SEM. Data were analyzed by one-way analysis of variance: * *p* < 0.05 and **** *p* < 0.0001 vs CTRL; *n* = 8.

The results showed no statistical effects of either B97% or B85% on any of the concentrations tested compared to the control cells; in contrast, treatment with H_2_O_2_ was able to induce ROS production by 1.45-fold with respect to untreated cells (one-way ANOVA: *p* < 0.0001 vs. CTRL) (Figure 8).

These data revealed no effects of BER or PROTBERs in inducing oxidative stress through ROS production. 

### 3.8. Transcriptional Effects of Berberis aristata Bark Extracts on Target Genes Involved in Cell Cycle Control and Neoplastic Transformation 

With the purpose of deepening the study on the safety and biological activity of BER, we investigated the molecular effects of BER- and PROTBERs-containing herbal preparations in an In Vitro model of human normal kidney cells, HEK293.

We focused on the transcriptional effects on the main target genes involved in the control of cell cycle, cell growth, neoplastic transformation, and oncogenes by exposing HEK293 cells for 24 h to the two examined extracts, B97% and B85%, at the concentrations of 10 and 20 µg/mL. First, we analyzed through RT-qPCR the gene expression of the tumor protein p53 (*TP53*), the oncogene *MDM2*, and the proto-oncogenes *c-MYC*, *n-MYC*, *HRAS*, and *MET*.

**Figure 9:**
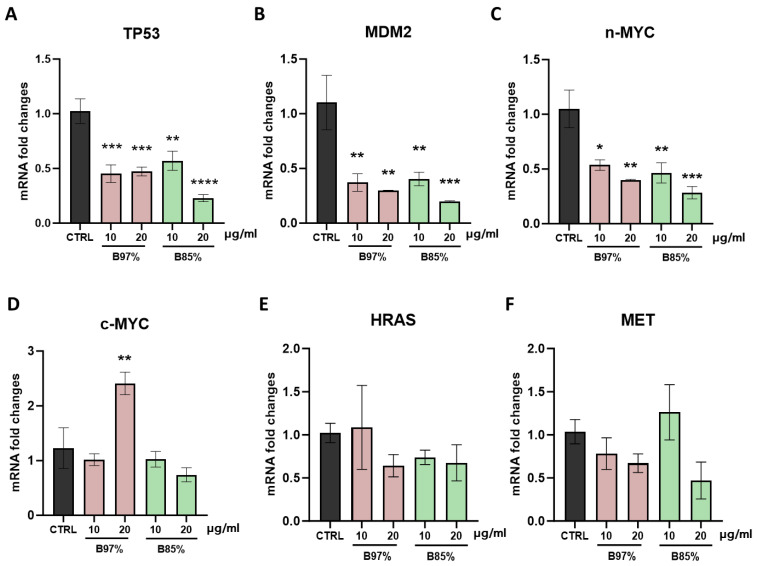
RT-qPCR analysis of *TP53* (**A**), *MDM2* (**B**), *n-MYC* (**C**), *c-MYC* (**D**), *HRAS* (**E**), and *MET* (**F**) transcripts in HEK293 cells following treatment with B97% and B85% at 10 or 20 μg/mL. Each column represents mean ± SEM. Data were analyzed by one-way analysis of variance followed by Dunnet post-hoc: * *p* < 0.05, ** *p* < 0.01, *** *p* < 0.001, and **** *p* < 0.0001 vs. CTRL (*n* = 4).

Our results showed that *TP53*, *MDM2*, and *n-MYC* mRNA levels were markedly downregulated in cells treated with B97% and B85% at both 10 and 20 µg/mL compared to the control counterpart [one-way ANOVA: *TP53*: F (4, 18) = 12.04, *p* < 0.001; *MDM2*: F (4, 18) = 7.773, *p* = 0.0008; *n-MYC:* F (4, 18) = 8.198, *p* = 0.0006] (Figure 9A–C). On the contrary, no differences were observed in *c-MYC* gene expression with respect to untreated cells with the exception of cells exposed to B97% at 20 µg/mL where a significant increase (2-fold) in *c-MYC* mRNA levels, compared to the control, was observed [F (4, 18) = 7.375; *p* = 0.0011] (Figure 9D). Regarding the mRNA levels of *HRAS* and *MET*, we found no significant effects of either BER extracts, at either concentration, in kidney cells following 24 h of treatment (Figure 9E,F). These results revealed the impact of both *B. aristata* extracts in downregulating the gene transcription of *TP53*, *MDM2*, and *n-MYC* at both the concentrations tested in normal cells. 

**Figure 10:**
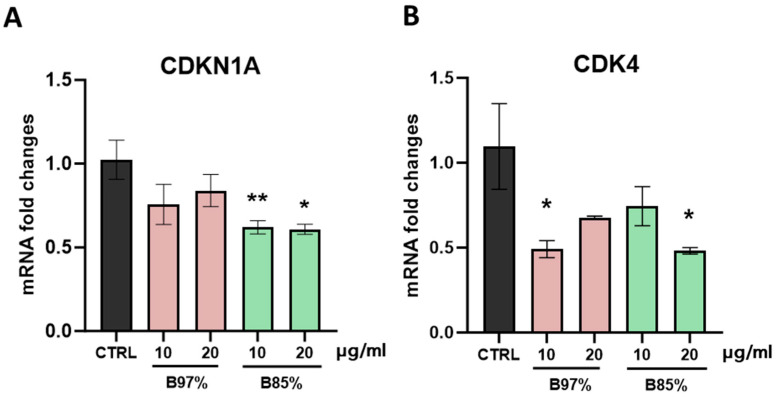
RT-qPCR analysis of *CDKN1A* (**A**) and *CDK4* (**B**) transcripts in HEK293 cells following treatment with B97% and B85% at 10 or 20 μg/mL. Each column represents mean ± SEM. Data were analyzed by one-way analysis of variance followed by Dunnet post-hoc: * *p* < 0.05 and ** *p* < 0.01 vs. CTRL (*n* = 4).

Then, our molecular analysis focused on p21 (*CDKN1A*) and *CDK4* targets, two essential factors in regulating cell cycle. The results revealed no statistically significant effects of the B97% extract on modulating *CDKN1A* gene transcription that was downregulated instead by B85% at both concentrations [F (4, 18) = 4.273, *p* = 0.0132]. Regarding *CDK4*, treatment with both BER extracts induced a decrease in gene expression unrelated to the dose [F (4, 18) = 3.036, *p* = 0.0446] (Figure 10A,B). 

In evaluating the effects of BER in regulating cell death processes, such as apoptosis, we also analyzed the transcriptional levels of the Bcl-2 family members, key factors in the early stages of apoptotic process: the pro-apoptotic gene *BAX* and the anti-apoptotic gene *BCL-2* [66,67,68]. 

**Figure 11:**
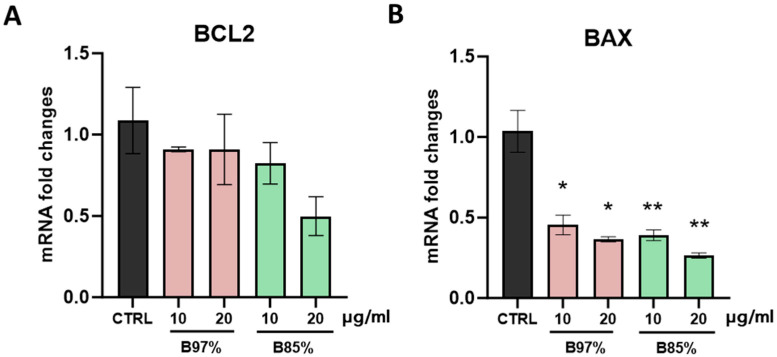
RT-qPCR analysis of *BAX* (**A**) and *BCL-2* (**B**) transcripts in HEK293 cells following treatment with B97% and B85% at 10 or 20 μg/mL. Each column represents mean ± SEM. Data were analyzed by one-way analysis of variance followed by Dunnet post-hoc: * *p* < 0.05 and ** *p* < 0.01 vs. CTRL (*n* = 4).

Statistical analysis showed a significant and similar effect of both *B. aristata* extracts in downregulating the expression of the pro-apoptotic gene *BAX* in HEK293 cells compared to controls [one-way ANOVA: F (4, 9) = 8.688, *p* = 0.0037], whereas no changes in *BCL-2* mRNA levels were observed (Figure 11A,B).

Accumulating evidence has revealed that BER exerts potential pro-apoptotic effects by modulating *BAX*/*BCL2* (pro-/anti-apoptotic) expression in multiple cancers, including breast, lung, liver, gastric, colorectal, pancreatic, and ovarian cancers [12,60,69,70,71,72,73,74]. Our results in normal cells counteracted this evidence and highlighted a *BAX* transcriptional inhibition. 

Overall, our data showed that both *B. aristata* extracts examined did not affect or downregulated the transcription of several gene targets involved in the regulation of cell cycle or with oncogenic functions, suggesting an inhibitory activity of BER in cell cycle, proliferation, and division processes in normal cells.

## 4. Discussion

Herbal preparations containing berberine (BER) and other minor protoberberinoids (PROTBERs) have been extensively used in traditional Asian medicine, in modern conventional phytotherapy, and in food supplements supporting physiological activities. BER has garnered attention for its purported effects on glucose and lipid metabolism, gastrointestinal health, and even for potential anticancer properties [11,15,16,17,75,76]. However, as the popularity of herbal preparations containing BER and PROTBERs increase, concerns about their biological safety are emerging, especially regarding the safe exposure for general consumers and special population, as highlighted by some national authorities and EFSA in the EU [37]. The authorities have clearly claimed the need for analytical, pharmacokinetic, and biological insights into herbal preparations containing BER and PROTBERs used as food supplements. This study aimed to investigate these important concerns to provide valuable knowledge to the authorities, the scientific community, and companies producing BER-based preparations. 

The chemical characterization of *B. aristata* crude extracts available in the market (the most used preparations containing BER and PROTBERs used in food supplements) allowed to confirm the declared content of BER and the identification of jatrorrhizine as the main secondary PROTBER, and palmatine and berberrubine as other minority PROTBERs present in the extracts.

These findings showed that herbal preparations containing BER and PROTBERs are more homogeneous than expected within the EU, being basically referred to as *B. aristata* dry extracts; moreover, BER represents more than 90% of total alkaloids in the most common extracts available in the market.

Pivotal evidence about the pharmacokinetic properties of BER have emerged from In Vitro assays and In Silico predictions; BER and PROTBERs are only minimally degraded before intestinal absorption, but being a P-gp substrate and metabolized by cytochrome P450 (CYP450), with particular regard to the CYP2D6, CYP1A2, and CYP3A4 isoforms, a low bioavailability is expected in particular for BER [35,52,61]. Computational predictions were confirmed experimentally for BER, and in part for palmatine, whereas a mainly structural-homology-based prediction could be obtained for jatrorrhizine and berberrubine. Besides LDLR, well known to be peculiar target of BER [77], all computational tools confirmed the activity of BER and PROTBERs towards neuroprotective [55,62,64,78,79] and antiproliferative targets [59,60,61,80,81].

These data allowed us to move towards a rational and deeper In Vitro safety assessment of the plausible concentrations of BER and PROTBERs that are able to reach different organism districts after oral administration. Noticeably, the target prediction and cell and molecular analysis addressed in this study were focused on human models, recognizing the limitations and misleading interpretations in extrapolating findings from animal studies to human safety [71]. Therefore, given the homogenous phytochemical profile available, we evaluated the effects of two representative *B. aristata* bark dry extracts on gastric (AGS), intestinal (Caco-2), hepatic (HepG2), and renal (HEK293) cells, following short- or a long-term exposure (4 and 24 h). The results for both exposures showed a very limited impact of BER-enriched extracts on cell viability, with an IC_50_ always exceeding 100 µg/mL in all cell lines examined. We considered cell viability only as a preliminary but fundamental test to assess the safe concentrations of samples under investigation, allowing us to quickly move towards the investigation of the interaction effects between BER and PROTBERs and CYP1A2 -2D6 and -3A4 substrates on hepatic cell viability. Interestingly, even at 50 and 100 µg/mL, the *B. aristata* bark dry extracts did not affect cell viability in the presence of phenacetin (CYP1A2), dextromethorphan (CYP2D6), or triazolam (CYP3A4), demonstrating that herbal preparations containing BER and PROTBERs may not have negative interactions with CYP450 substrates.

Many In Vitro studies demonstrated the ability of BER to inhibit cell proliferation, invasion, and to regulate the cell cycle in tumoral cells [59,61,80,81,82,83]. In light of this, our study proceeded with the analysis of cell response to the exposure to both BER extracts by evaluating the cell migration of colorectal adenocarcinoma cells (Caco-2) through the wound-healing assay. This test is a commonly used method in cell biology to determine the effects of compounds on the changes in migration properties, an important feature of cells to move and invade other tissues, interact with other cells, or to repair any damage. This property is typical of cancer cells and identifies their invasive and aggressive potential. Our data demonstrated a delay in wound closure when treated with *B. aristata* bark dry extracts, even after a long exposure time, both at 10 and 100 µg/mL, corroborating numerous evidence regarding the BER and PROTBER inhibitory effects of cell migration [80,81,84,85,86]. Our experimental evidence supported the potential antitumoral activity of *B. aristata* extracts.

Subsequently, the study focused on a non-tumor cell model in order to gather preliminary data on the possible cell function alterations induced by *B. aristata* extracts, responsible for the transformation from normal to tumor cell. Among the mechanisms involved in neoplastic transformation, the irreversible damage to DNA and a dysregulation of the cell cycle are the first to appear. In addition, the excessive generation of intracellular ROS in response to oxidative stress may lead to DNA damage and mutagenesis through the suppression and mutation of tumor suppressor genes. This phenomenon potentially promotes tumorigenesis [87,88]. The *B. aristata* extracts studied did not induce ROS production in human normal HEK293 cells, thus excluding the hypothesis of a potential effect of DNA damage mediated by excess free radicals. These data supported evidence reporting BER antioxidant activity by suppressing ROS production [89,90,91,92]. Results confirming a non-carcinogenic potential of BER also emerged from the molecular analysis in normal HEK293 cells through the evaluation of BER modulatory effects on the expression of oncogenes (*MDM2*, *MYC family*, *HRAS*, *MET*), or key genes in the control of cell cycle phases (*TP53*, *CDKN1A*, *CDK4*) or the apoptosis process (*BAX*, *BCL2*). Overall, with the exception of *c-MYC* expression being slightly upregulated by B97 20 µg/mL, a general downregulation of cell cycle genes was observed, highlighting the inhibitory effect of *B. aristata* extracts in promoting the cell cycle, and thus cell proliferation in normal cells [59,83]; moreover, no effects on the transcription of proto-oncogenes emerged, supporting data on the absence of the pro-tumoral potential of BER [16,93,94,95]. In addition, both the *B. aristata* extracts investigated were able to downregulate the expression of the pro-apoptotic gene *BAX*, suggesting the effects of BER in regulating at transcriptional level the apoptosis process, having no impact on apoptosis-mediated cell death in normal cells different to what was demonstrated in tumor cells [68,69,73,96]. These preliminary findings highlighted important insights into BER and PROTBERs safety in normal cells considering the crucial role of these target genes in controlling cell functions and cell homeostasis.

Summarizing, our findings allowed us to determine (i) the chemical characterization and quantification of BER and minor PROTBERs in the most marketed raw materials for food supplements; (ii) new knowledge about the pharmacokinetic and bioaccessibility properties of BER and minor PROTBERs; (iii) and finally, preliminary data on biological safety in terms of cytotoxicity and pro- or antitumoral activity.

Although the need for more research and insights into biological activity, data emerged from this study provide a broad-spectrum analysis of the most common *B. aristata* bark extracts. Overall, this evidence may represent a relevant update to better elucidate the critical points raised by the authorities related to the safety evaluation of BER-containing herbal preparations used in food supplements.

## 5. Conclusions

This work presents several notable strengths that deserve to be highlighted:

(i) The study addresses the bioaccessibility and safety of berberine and protoberberinoids through a highly transferrable conceptual framework. It focuses on the most widely recognized and utilized extracts available in the food supplement market, which have been chemically characterized;
(ii) Integrated In Silico and In Vitro approaches provide valuable insights. The study concurrently evaluates the bioaccessibility of the examined samples, the impact of *Berberis aristata* extracts on hepatic toxicity in the presence of CYP450 substrates, the effects of cell toxicity and cell migration on tumor cells, and the potential pro-oxidant and pro-tumoral effects in non-tumoral human cells;
(iii) The protocol employed considers different exposure times and treatment concentrations, enhancing the robustness of the In Vitro models used.

However, we aware of certain limitations of the study:

(i) In future research, we intend to expand the pharmacokinetic evaluation of the major alkaloids contained in *B. aristata* extracts, including dynamic cell absorption models;
(ii) While the current focus was on gene expression and transcriptional response to investigate early cellular response, we plan to explore the impact of berberine and protoberberinoids on human cell lines by examining upstream and downstream cell signaling pathways and protein levels related to antiproliferative activity;
(iii) Finally, we are fully aware that our findings are derived solely from In Vitro tests, and further confirmation through additional studies is necessary.

## Figures and Tables

**Table 1:** Transcript and sequence of each primer used in real-time PCR.

Gene	NCBI GenBank	Sequence
*Rplp13a*	NM_012423.4	fw: GTGCGTCTGAAGCCTACAAG rv: CGTTCTTCTCGGCCTGTTTC
*Rps18*	NM_022551.3	fw: TCTAGTGATCCCTGAAAAGT rv: AACACCACATGAGCATATC
*Tp53*	NM_000546.6	fw: AGGGATGTTTGGGAGATGTAAG rv: CCTGGTTAGTACGGTGAAGTG
*c-Myc*	NM_001354870.1	fw: AAGCTGAGGCACACAAAGA rv: GCTTGGACAGGTTAGGAGTAAA
*n-Myc*	NM_005378.6	fw: TCCAGCAGATGCCACATAAG rv: ACCTCTCATTACCCAGGATGTA
*Met*	NM_001127500.3	fw: CCTGGGCACCGAAAGATAAA rv: CTCCTCTGCACCAAGGTAAAC
*Mdm2*	NM_002392.6	fw: AGGCTGATCTTGAACTCCTAAAC rv: CAGGTGCCTCACATCTGTAATC
*Cdkn1a*	NM_000389.5	fw: CGGAACAAGGAGTCAGACATT rv: AGTGCCAGGAAAGACAACTAC
*Snai1*	NM_005985.4	fw: CAGATGAGGACAGTGGGAAAG rv: GAGACTGAAGTAGAGGAGAAGGA
*Snai2*	NM_003068.5	fw: AACTACAGCGAACTGGACAC rv: GAGGATCTCTGGTTGTGGTATG
*Hras*	NM_005343.4	fw: AAGCAAGGAAGGAAGGAAGG rv: GTGGCATTTGGGATGTTCAAG
*Cdk4*	NM_000075.4	fw: GCTCTGCAGCACTCTTATCTAC rv: CTCAGTGTCCAGAAGGGAAATG
*Bax*	NM_004324	fw: CTCCCCATCTTCAGATCATCAG rv: GGCAGAAGGCACTAATCAAGTC
*Bcl2*	NM_000657	fw: GACTGAGTACCTGAACCGGC rv: CTCAGCCCAGACTCACATCA

**Table 2:** Parameters of HPLC-DAD method employed.

Parameter	Value
R^2^	0.99
Equation	y = 4317.80 − 98.57
Linearity range	0.04–7.5 μg in column
Recovery of spiked standard	>90% and <105%
Limit of quantification	0.03 μg in column
Intra- and inter-day variation	<3%

**Table 3:** Quantification of berberine in 16 different marketed herbal preparations containing berberine and protoberberinoids by means of HPLC-DAD. Quantification was made on dry basis (db) of the extracts, and values are expressed as berberine hydrochloride. Values are expressed as % *w*/*w* of a triplicate analysis performed on eight *B. aristata* extracts with a declared content of 97% of berberine hydrochloride db and on eight extracts of *B. aristata* with a declared content of 85% of berberine hydrochloride.

Sample	Berberine % *w*/*w* db	Sample	Berberine % *w*/*w* db
A85	86.26	A97	97.41
B85	86.74	B97	91.94
C85	91.93	C97	96.50
D85	88.87	D97	97.16
E85	89.63	E97	97.49
F85	90.66	F97	97.21
G85	80.07	G97	97.29
J85	87.88	J97	97.80
mean ± SD	87.76 ± 3.64	mean ± SD	96.60 ± 1.91

**Table 4:** Quantification of protoberberinoids in *B. aristata* bark extracts. Samples are labeled according to the declared content of berberine expressed as berberine hydrochloride. Quantification was made on dry basis (db) of the extracts and all compounds are expressed as berberine hydrochloride. Values are expressed as % *w*/*w* of a triplicate analysis performed on eight *B. aristata* extracts with a declared content of 97% of berberine hydrochloride db and on eight extracts of *B. aristata* with a declared content of 85% of berberine hydrochloride. The quantification of palmatine and berberrubine was performed by repeating chromatographic runs of high amounts of injected samples in order to reach the limit of quantification.

Samples	Jatrorrhizine % *w*/*w* db	Berberrubine + Palmatine % *w*/*w* db
A85	2.39	0.25
B85	1.88	0.24
C85	2.10	0.23
D85	3.07	0.26
E85	2.54	0.28
F85	2.85	0.24
G85	2.59	0.20
J85	3.12	0.25
mean ± SD	2.57 ± 0.44	0.24 ± 0.02
A97	2.25	0.24
B97	1.49	0.08
C97	2.98	0.11
D97	2.57	0.23
E97	2.04	0.13
F97	2.04	0.14
G97	2.05	0.28
J97	1.71	0.13
mean ± SD	2.14 ± 0.46	0.17 ± 0.07

**Table 5:** Chemical composition of pooled A85 and D85 (mixed 1:1, B85) and pooled A97 and G97 (1:1, B97) expressed as mean of composition of single extracts.

Samples	Berberine % *w*/*w* db	Jatrorrhizine % *w*/*w* db	Berberrubine + Palmatine % *w*/*w* db
*B. aristata* 85% (B85)	87.57 ± 1.85	2.83 ± 0.48	0.26 ± 0.01
*B. aristata* 97% (B97)	97.35 ± 0.08	2.15 ± 0.14	0.26 ± 0.02

**Table 6:** Bioaccessibility rate of berberine and jatrorrhizine investigated in two extracts of *Berberis aristata* DC. bark containing 97% or 85% of berberine hydrochloride dry basis, respectively. Berberine showed high stability in response to pH change and digestive enzyme activity; jatrorrhizine was also recovered in high percentage.

	Bioaccessibility Rate %	
Samples	Berberine	Jatrorrhizine
*B. aristata* 97%	>95	83.57 ± 3.33
*B. aristata* 85%	>95	87.81 ± 3.69

**Table 7:** IC50 values calculated for the extracts of *B. aristata* bark containing berberine hydrochloride 97% (B97) and 85% (B85) by means cell viability assay performed on intestinal (Caco-2), gastric (AGS), hepatic (HepG2), and kidney (HEK293) cells after 4 h and 24 h of treatment.

			IC_50_ (µg/mL)		
Sample	Treatment (h)	AGS	Caco-2	HepG2	HEK293
B97%	4	>200	166.93 ± 8.44	>200	142.65 ± 12.01
B85%	4	>200	169.14 ± 9.25	>200	181.93 ± 24.50
B97%	24	>200	105.59 ± 11.21	198.85 ± 8.50	127.16 ± 25.18
B85%	24	>200	107.34 ± 9.68	186.41 ± 8.42	143.43 ± 29.80

Data are expressed in µg/mL as mean ± standard deviation (SD).

## Data Availability

Data are available upon request to the Corresponding author upon reasonable request. Data are currently being assessed by EFSA.

## Supplementary Materials

The following supporting information can be downloaded at: https://www.mdpi.com/article/10.3390/nu16172953/s1, Figure S1: Computational analysis of jatrorrhizine (A), berberrubine (B), and palmatine (C) pharmacokinetic properties by means of SwissADME tool (http://www.swissadme.ch/); Figure S2: Genecards targets prediction for the interaction between berberine and jatrorrhizine (A), berberine and berberrubine (B), and berberine and palmatine (C) respectively; Figure S3: Analysis of target prediction by means of SwissTarget Prediction (STP) and SEA tools for berberine (A), jatrorrhizine (B), berberrubine (C), and palmatine (D).
