# Supplementary material for: Comprehensive Analysis of Berberis aristata DC. Bark Extracts: In Vitro and In Silico Evaluation of Bioaccessibility and Safety
Source: Nutrients. 2024 Sep 2;16(17):2953. doi: 10.3390/nu16172953 (PMC11397700; doi:10.3390/nu16172953)
Supplement: Supplementary file 1 [file nutrients-16-02953-s001.zip › nutrients-3158862-supplementary.pdf]

Supplementary figure S1

A

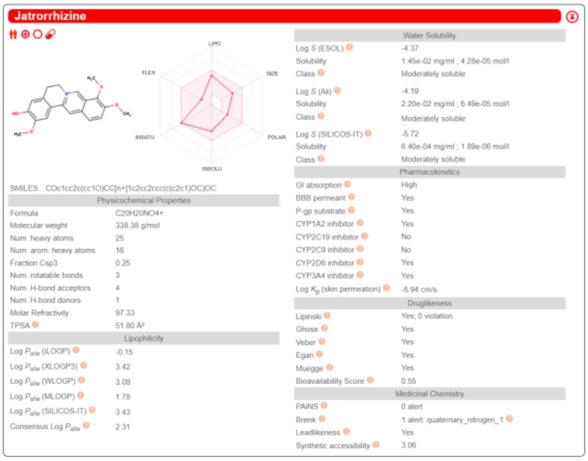

Physicochemical Properties	
Formula	C20H20NO4+
Molecular weight	338.38 g/mol
Num. heavy atoms	25
Num. arom. heavy atoms	16
Fraction Csp3	0.25
Num. rotatable bonds	3
Num. H-bond acceptors	4
Num. H-bond donors	1
Molar Refractivity	97.33
TPSA	51.80 Å²
**Lipophilicity**	
Log $P_{o/w}$ (iLOGP)	-0.15
Log $P_{o/w}$ (XLOGP3)	3.42
Log $P_{o/w}$ (WLOGP)	3.08
Log $P_{o/w}$ (MLOGP)	1.78
Log $P_{o/w}$ (SILICOS-IT)	3.43
Consensus Log $P_{o/w}$	2.31

Water Solubility	
Log *S* (ESOL)	-4.37
Solubility	1.45e-02 mg/ml ; 4.28e-05 mol/l
Class	Moderately soluble
Log *S* (Ali)	-4.19
Solubility	2.20e-02 mg/ml ; 6.49e-05 mol/l
Class	Moderately soluble
Log *S* (SILICOS-IT)	-5.72
Solubility	6.40e-04 mg/ml ; 1.89e-06 mol/l
Class	Moderately soluble
**Pharmacokinetics**	
GI absorption	High
BBB permeant	Yes
P-gp substrate	Yes
CYP1A2 inhibitor	Yes
CYP2C19 inhibitor	No
CYP2C9 inhibitor	No
CYP2D6 inhibitor	Yes
CYP3A4 inhibitor	Yes
Log K_p (skin permeation)	-5.94 cm/s
**Druglikeness**	
Lipinski	Yes; 0 violation
Ghose	Yes
Veber	Yes
Egan	Yes
Muegge	Yes
Bioavailability Score	0.55
**Medicinal Chemistry**	
PAINS	0 alert
Brenk	1 alert: quaternary_nitrogen_1
Leadlikeness	Yes
Synthetic accessibility	3.06

B

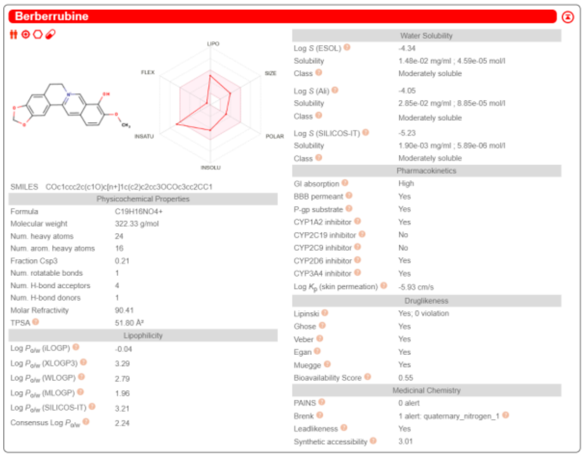

Physicochemical Properties	
Formula	C19H16NO4+
Molecular weight	322.33 g/mol
Num. heavy atoms	24
Num. arom. heavy atoms	16
Fraction Csp3	0.21
Num. rotatable bonds	1
Num. H-bond acceptors	4
Num. H-bond donors	1
Molar Refractivity	90.41
TPSA	51.80 Å²
**Lipophilicity**	
Log $P_{o/w}$ (iLOGP)	-0.04
Log $P_{o/w}$ (XLOGP3)	3.29
Log $P_{o/w}$ (WLOGP)	2.79
Log $P_{o/w}$ (MLOGP)	1.96
Log $P_{o/w}$ (SILICOS-IT)	3.21
Consensus Log $P_{o/w}$	2.24

Water Solubility	
Log *S* (ESOL)	-4.34
Solubility	1.48e-02 mg/ml ; 4.59e-05 mol/l
Class	Moderately soluble
Log *S* (Ali)	-4.05
Solubility	2.85e-02 mg/ml ; 8.85e-05 mol/l
Class	Moderately soluble
Log *S* (SILICOS-IT)	-5.23
Solubility	1.90e-03 mg/ml ; 5.89e-06 mol/l
Class	Moderately soluble
**Pharmacokinetics**	
GI absorption	High
BBB permeant	Yes
P-gp substrate	Yes
CYP1A2 inhibitor	Yes
CYP2C19 inhibitor	No
CYP2C9 inhibitor	No
CYP2D6 inhibitor	Yes
CYP3A4 inhibitor	Yes
Log K_p (skin permeation)	-5.93 cm/s
**Druglikeness**	
Lipinski	Yes; 0 violation
Ghose	Yes
Veber	Yes
Egan	Yes
Muegge	Yes
Bioavailability Score	0.55
**Medicinal Chemistry**	
PAINS	0 alert
Brenk	1 alert: quaternary_nitrogen_1
Leadlikeness	Yes
Synthetic accessibility	3.01

C

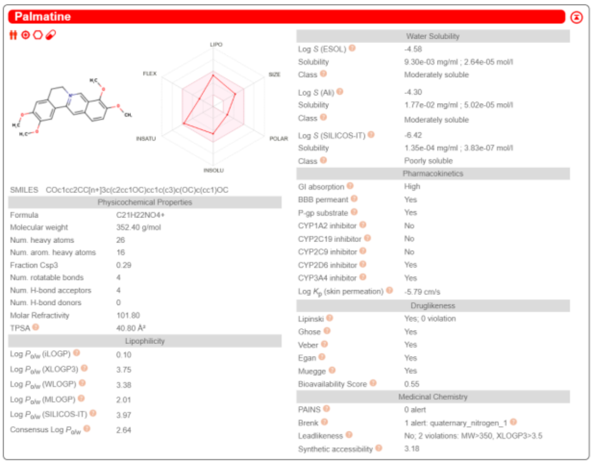

Physicochemical Properties	
Formula	C21H22NO4+
Molecular weight	352.40 g/mol
Num. heavy atoms	26
Num. arom. heavy atoms	16
Fraction Csp3	0.29
Num. rotatable bonds	4
Num. H-bond acceptors	4
Num. H-bond donors	0
Molar Refractivity	101.80
TPSA	40.80 Å²
**Lipophilicity**	
Log $P_{o/w}$ (iLOGP)	0.10
Log $P_{o/w}$ (XLOGP3)	3.75
Log $P_{o/w}$ (WLOGP)	3.38
Log $P_{o/w}$ (MLOGP)	2.01
Log $P_{o/w}$ (SILICOS-IT)	3.97
Consensus Log $P_{o/w}$	2.64

Water Solubility	
Log *S* (ESOL)	-4.58
Solubility	9.30e-03 mg/ml ; 2.64e-05 mol/l
Class	Moderately soluble
Log *S* (Ali)	-4.30
Solubility	1.77e-02 mg/ml ; 5.02e-05 mol/l
Class	Moderately soluble
Log *S* (SILICOS-IT)	-6.42
Solubility	1.35e-04 mg/ml ; 3.83e-07 mol/l
Class	Poorly soluble
**Pharmacokinetics**	
GI absorption	High
BBB permeant	Yes
P-gp substrate	Yes
CYP1A2 inhibitor	No
CYP2C19 inhibitor	No
CYP2C9 inhibitor	No
CYP2D6 inhibitor	Yes
CYP3A4 inhibitor	Yes
Log K_p (skin permeation)	-5.79 cm/s
**Druglikeness**	
Lipinski	Yes; 0 violation
Ghose	Yes
Veber	Yes
Egan	Yes
Muegge	Yes
Bioavailability Score	0.55
**Medicinal Chemistry**	
PAINS	0 alert
Brenk	1 alert: quaternary_nitrogen_1
Leadlikeness	No; 2 violations: MW>350, XLOGP3>3.5
Synthetic accessibility	3.18

Computational analysis of jatrorrhizine (A), berberrubine(B), and palmatine (C) pharmacokinetic properties by means of SwissADME tool (http://www.swissadme.ch/).

Supplementary figure S2

A

Showing 6 of 6 results for berberine AND jatrorrhizine Search Time: 0 ms in All sections (18)

(Click on the icon in the table below to see search hit context)

Export | Show: 25

	Symbol	Description	Category	UniProt ID	GIFtS	GC id	Score
1	LINC01672	Long Intergenic Non-Protein Coding RNA 1672	RNA Gene		13	GC01P008900	7.09
2	BDNF-AS	BDNF Antisense RNA	RNA Gene		22	GC11P027466	7.07
3	TMX2-CTNND1	TMX2-CTNND1 Readthrough (NMD Candidate)	RNA Gene		17	GC11P057712	5.03
4	RAD52	RAD52 Homolog, DNA Repair Protein	Protein Coding	P43351	45	GC12M000912	4.51
5	SCARNA5	Small Cajal Body-Specific RNA 5	RNA Gene		21	GC02P233275	4.26
6	ACHE	Acetylcholinesterase (Cartwright Blood Group)	Protein Coding	P22303	50	GC07M100889	1.66

1 << < 1 > >> 1

B

Showing 9 of 9 results for berberine AND berberrubine Search Time: 0 ms in All sections (18)

(Click on the icon in the table below to see search hit context)

Export | Show: 25

	Symbol	Description	Category	UniProt ID	GIFtS	GC id	Score
1	TNF	Tumor Necrosis Factor	Protein Coding	P01375	55	GC06P118895	7.83
2	LINC01672	Long Intergenic Non-Protein Coding RNA 1672	RNA Gene		13	GC01P008900	7.19
3	ABCC8	ATP Binding Cassette Subfamily C Member 8	Protein Coding	Q09428	50	GC11M017392	6.42
4	KCNJ11	Potassium Inwardly Rectifying Channel Subfamily J Member 11	Protein Coding	Q14654	50	GC11M017865	6.06
5	TOP1	DNA Topoisomerase I	Protein Coding	P11387	52	GC20P041028	5.58
6	CCL2	C-C Motif Chemokine Ligand 2	Protein Coding	P13500	52	GC17P034255	4.78
7	IL1B	Interleukin 1 Beta	Protein Coding	P01584	51	GC02M112829	4.76
8	CXCL8	C-X-C Motif Chemokine Ligand 8	Protein Coding	P10145	47	GC04P073740	4.62
9	SNORD55	Small Nucleolar RNA, C/D Box 55	RNA Gene		18	GC01P045864	2.69

C

Showing 15 of 15 results for berberine AND palmatine Search Time: 0 ms in All sections (18)

(Click on the icon in the table below to see search hit context)

Export | Show: 25

	Symbol	Description	Category	UniProt ID	GIFtS	GC id	Score
1	LINC01672	Long Intergenic Non-Protein Coding RNA 1672	RNA Gene		13	GC01P008900	8.61
2	BDNF-AS	BDNF Antisense RNA	RNA Gene		22	GC11P027466	8.22
3	CYP1A2	Cytochrome P450 Family 1 Subfamily A Member 2	Protein Coding	P05177	48	GC15P074748	5.90
4	RAD52	RAD52 Homolog, DNA Repair Protein	Protein Coding	P43351	45	GC12M000912	5.75
5	TP53	Tumor Protein P53	Protein Coding	P04637	57	GC17M007661	5.29
6	TMX2-CTNND1	TMX2-CTNND1 Readthrough (NMD Candidate)	RNA Gene		17	GC11P057712	4.90
7	AR	Androgen Receptor	Protein Coding	P10275	55	GC0XP067544	4.73
8	SCARNA5	Small Cajal Body-Specific RNA 5	RNA Gene		21	GC02P233275	4.23
9	CYP1A1	Cytochrome P450 Family 1 Subfamily A Member 1	Protein Coding	P04798	51	GC15M074719	3.72
10	AHR	Aryl Hydrocarbon Receptor	Protein Coding	P35869	50	GC07P016916	3.72
11	ABCB1	ATP Binding Cassette Subfamily B Member 1	Protein Coding	P08183	54	GC07M087504	3.54
12	BCHE	Butyrylcholinesterase	Protein Coding	P06276	53	GC03M165772	3.23
13	SOD1	Superoxide Dismutase 1	Protein Coding	P00441	56	GC21P031659	2.06
14	CAT	Catalase	Protein Coding	P04040	55	GC11P034460	2.06
15	BACE1	Beta-Secretase 1	Protein Coding	P56817	51	GC11M117285	0.44

Genecards targets prediction for the interaction between berberine and jatrorrhizine (A), berberine and berberrubine (B) and berberine and palmatine (C) respectively. (https://www.genecards.org/).

Supplementary figure S3

A

Target	Common name	Uniprot ID	ChEMBL ID	Target Class	Probability*	Known actives (3D/2D)
Acetylcholinesterase	ACHE	P22303	CHEMBL220	Hydrolase		109 / 19
Serotonin 2b (5-HT2b) receptor	HTR2B	P41595	CHEMBL1833	Family A G protein-coupled receptor		17 / 1
Butyrylcholinesterase	BCHE	P06276	CHEMBL1914	Hydrolase		23 / 7
Adrenergic receptor alpha-2	ADRA2C	P18825	CHEMBL1916	Family A G protein-coupled receptor		2 / 1
Alpha-2b adrenergic receptor	ADRA2B	P18089	CHEMBL1942	Family A G protein-coupled receptor		3 / 1
Muscarinic acetylcholine receptor M1	CHRM1	P11229	CHEMBL216	Family A G protein-coupled receptor		28 / 1
Sigma opioid receptor	SIGMAR1	Q99720	CHEMBL287	Membrane receptor		33 / 3
Cytochrome P450 2D6	CYP2D6	P10635	CHEMBL289	Cytochrome P450		3 / 1
SUMO-activating enzyme	SAE1 UBA2	Q9UBE0 Q9UBT2	CHEMBL2095174	Enzyme		4 / 1
Ras-related C3 botulinum toxin substrate 1	RAC1	P63000	CHEMBL6094	Unclassified protein		0 / 1
Cell division control protein 42 homolog	CDC42	P60953	CHEMBL6088	Unclassified protein		1 / 1

B

Target	Common name	Uniprot ID	ChEMBL ID	Target Class	Probability*	Known actives (3D/2D)
Acetylcholinesterase	ACHE	P22303	CHEMBL220	Hydrolase		92 / 21
Butyrylcholinesterase	BCHE	P06276	CHEMBL1914	Hydrolase		19 / 9
Serotonin 2b (5-HT2b) receptor	HTR2B	P41595	CHEMBL1833	Family A G protein-coupled receptor		23 / 1
Adrenergic receptor alpha-2	ADRA2C	P18825	CHEMBL1916	Family A G protein-coupled receptor		6 / 1
Alpha-2b adrenergic receptor	ADRA2B	P18089	CHEMBL1942	Family A G protein-coupled receptor		5 / 1
Muscarinic acetylcholine receptor M1	CHRM1	P11229	CHEMBL216	Family A G protein-coupled receptor		26 / 1
Sigma opioid receptor	SIGMAR1	Q99720	CHEMBL287	Membrane receptor		202 / 4
Cytochrome P450 2D6	CYP2D6	P10635	CHEMBL289	Cytochrome P450		4 / 1
SUMO-activating enzyme	SAE1 UBA2	Q9UBE0 Q9UBT2	CHEMBL2095174	Enzyme		9 / 1
Cell division control protein 42 homolog	CDC42	P60953	CHEMBL6088	Unclassified protein		1 / 1
Ras-related C3 botulinum toxin substrate 1	RAC1	P63000	CHEMBL6094	Unclassified protein		1 / 1

C

Target	Common name	Uniprot ID	ChEMBL ID	Target Class	Probability*	Known actives (3D/2D)
Muscarinic acetylcholine receptor M1	CHRM1	P11229	CHEMBL216	Family A G protein-coupled receptor		0 / 1
Sigma opioid receptor	SIGMAR1	Q99720	CHEMBL287	Membrane receptor		0 / 3
Cytochrome P450 2D6	CYP2D6	P10635	CHEMBL289	Cytochrome P450		0 / 1
SUMO-activating enzyme	SAE1 UBA2	Q9UBE0 Q9UBT2	CHEMBL2095174	Enzyme		0 / 1
Butyrylcholinesterase	BCHE	P06276	CHEMBL1914	Hydrolase		1 / 7
Adrenergic receptor alpha-2	ADRA2C	P18825	CHEMBL1916	Family A G protein-coupled receptor		1 / 1
Alpha-2b adrenergic receptor	ADRA2B	P18089	CHEMBL1942	Family A G protein-coupled receptor		1 / 1
Acetylcholinesterase	ACHE	P22303	CHEMBL220	Hydrolase		1 / 19
Serotonin 2b (5-HT2b) receptor	HTR2B	P41595	CHEMBL1833	Family A G protein-coupled receptor		1 / 1
Cell division control protein 42 homolog	CDC42	P60953	CHEMBL6088	Unclassified protein		0 / 1
Ras-related C3 botulinum toxin substrate 1	RAC1	P63000	CHEMBL6094	Unclassified protein		0 / 1
Glyoxalase I	GLO1	Q04760	CHEMBL2424	Enzyme		8 / 0
Matrix metalloproteinase 12	MMP12	P39900	CHEMBL4393	Protease		1 / 0
Adenosine A2a receptor *(by homology)*	ADORA2A	P29274	CHEMBL251	Family A G protein-coupled receptor		15 / 0
Carbonic anhydrase II	CA2	P00918	CHEMBL205	Lyase		22 / 16

D

Target	Common name	Uniprot ID	ChEMBL ID	Target Class	Probability*	Known actives (3D/2D)
Acetylcholinesterase	ACHE	P22303	CHEMBL220	Hydrolase		129 / 22
Serotonin 2b (5-HT2b) receptor	HTR2B	P41595	CHEMBL1833	Family A G protein-coupled receptor		19 / 2
Butyrylcholinesterase	BCHE	P06276	CHEMBL1914	Hydrolase		40 / 8
Adrenergic receptor alpha-2	ADRA2C	P18825	CHEMBL1916	Family A G protein-coupled receptor		5 / 1
Alpha-2b adrenergic receptor	ADRA2B	P18089	CHEMBL1942	Family A G protein-coupled receptor		4 / 1
Muscarinic acetylcholine receptor M1	CHRM1	P11229	CHEMBL216	Family A G protein-coupled receptor		55 / 1
Sigma opioid receptor	SIGMAR1	Q99720	CHEMBL287	Membrane receptor		73 / 5
Cytochrome P450 2D6	CYP2D6	P10635	CHEMBL289	Cytochrome P450		2 / 1
SUMO-activating enzyme	SAE1 UBA2	Q9UBE0 Q9UBT2	CHEMBL2095174	Enzyme		10 / 1
Ras-related C3 botulinum toxin substrate 1	RAC1	P63000	CHEMBL6094	Unclassified protein		0 / 1
Cell division control protein 42 homolog	CDC42	P60953	CHEMBL6088	Unclassified protein		1 / 1
Dual specificity mitogen-activated protein kinase kinase 1	MAP2K1	Q02750	CHEMBL3587	Kinase		47 / 0

Analysis of target prediction by means of SwissTarget Prediction (STP) and SEA tools for berberine (A), jatrorrizine (B), berberrubine (C), and palmatine (D).
